# Foreign Correspondence

**Published:** 1889-05

**Authors:** 


### Foreign Correspondence.

To the Editor.—The following letter received from Dr. Miller may be of interest to your readers; if so, you are at liberty to use it.

Geo. S. Allen.

My Dear Dr. Allen.—I become daily more and more convinced that the explanation I have given of the phenomena of dental caries is the true one and that the inflammatory theory has not the slightest ground in fact, and has been imposed upon us now about long enough. I cannot state the grounds against the inflammatory theory any more clearly than I have in my various articles in the Independent Practitioner (especially the article in the April No., 1885). They are first the complete absence of all the cardinal symptoms of inflammation.

2. The absolute impossibility of producing a trace of caries by any of the methods which would cause inflammation of other living tissues of the human body.

The very operation of filling teeth is an argument against the inflammatory theory. If we were to bore a hole in the soft tissues or in bone, and hammer into it a plug of gold it would, in ninety-nine cases out of one hundred, be thrown off by suppuration. On the other hand, we can offer no insult of a mechanical nature to a tooth severe enough to produce caries.

3. Caries of dead dentine and artificial caries are perfectly analogous with caries of living dentine, a fact which the advocates of the inflammatory theory seem to delight in ignoring.

4. The microscopical appearances of carious dentine are not those seen in inflamed tissue.

5. There is no change in the structure of dentine where micro-organisms are not present. (This conclusion will be found in the Independent Practitioner for 1883.) That alone is sufficient to render the inflammatory theory untenable, and yet I know of no attempt's having been made to controvert it.

Wherever we have a dissolution of the basis substance, tubuli flow into each other, or caverns are formed by the destruction of a certain amount of the tissue, there we always find fungi and without fungi these changes do not and cannot take place.

We will pass over the old story of the predisposing causes, defective structure, bad development, deep fissures, crowded arch, etc., etc. That teeth in such conditions will yield more quickly to the forces of decay, every one knows and admits. It does not need much acumen to tell that a weak body yields more easily to a given force than a strong body. But *these things are not causes of decay.* If a jaw-bone containing the softest, worst developed, most crowded teeth imaginable were laid away in a box, the teeth would never decay; in the mouth they do decay. Now the question is: What agents are present in the mouth and not present in the box? Why do the teeth decay in one case and not in the other? I have time and again stated the various conditions of disease in which the saliva is said to have an acid reaction. I have mentioned the influence of acid foods and acid medicines.

I have called attention to the extensive destruction of the teeth inaugurated by a grape or lemon cure. I am perfectly mindful of all these agencies, but still it is not difficult to see that they are seldom causes of the caries we are daily called upon to treat. Such acids manifest their action upon the more exposed surfaces of the teeth, with which they readily come in contact (any one who has seen the effect of a long-continued grape or lemon cure knows that), and not upon those obscure points where true caries most frequently occur.

It is usual to speak of the acidity of the secretions of the mucous glands as an important factor in the production of caries of the neck, although I am not aware that any one has as yet demonstrated the frequency or strength of this acidity, or even directly proved that the fresh mucous is under any circumstances aciduous. Herrmann says: "Der schliem ist eine klare schlüpferige, fadenziehende, *alkahalische* Flüssigkeit," while Kirk (the physiologist), speaks of the mucous as an *acid* substance, so that until the question has been more carefully investigated from a dental standpoint, we have no right to call in an acidulous mucous to account for everything, of which the real cause may not be apparent.

Still less do I think that the serous exudation from irritated or inflamed gums can be looked upon as a cause of neck-caries. In the first place we have no reason to think that blood serum has any solvent or decalcifying action upon dentine. I subjected pulverized dentine to the action of blood serum for forty-eight hours, and found no lime salts in the filtrate.

In the second place, clinical observations do not appear to me to support this view. We constantly meet with cases of chronic inflammation of the gums, sometimes lasting for years, where the teeth remain intact, and it is notorious that in pyorrhœa alveolaris the teeth are rather less than usual subject to caries. Besides this, when we do find caries in these cases, we must not overlook the fact that the pockets at the margin of the gnms form recepticles for food, an unfailing factor in the production of caries.

I do not by any means wish to exclude an acid mucous or an acid exudation from inflamed gums; but I look upon the former as an unproved, and the latter as an impossible.

The dental profession is too much given to founding arguments upon suppositions and theories, and are everlastingly bringing forward views and presenting ideas without making the slightest attempt to test their truth experimentally. What we need is facts, and those who cannot present facts had better keep still.

It should be borne in mind that very many bacteria, whether putrefactive or fermentative, pathogenic or non-pathogenic, produce a ferment, which in its action is analogous to pepsin, *i. e.*, it has the power to dissolve albumen and albuminoid substances (to which group of substances deçalcified dentine belongs). The destruction of decalcified dentine takes place by solution (just as a piece of cooked egg is dissolved by many bacteria), and may be brought about by *any bacterium* which produces the ferment referred to.

Voss strasse, 32 Berlin. W. D. Miller.

